# Cell penetrating peptide (CPP) gold(iii) – complex – bioconjugates: from chemical design to interaction with cancer cells for nanomedicine applications†

**DOI:** 10.1039/d2na00096b

**Published:** 2022-05-12

**Authors:** Celia Arib, Audrey Griveau, Joel Eyer, Jolanda Spadavecchia

**Affiliations:** CNRS, UMR 7244, CSPBAT, Laboratoire de Chimie, Structures et Propriétés de Biomatériaux et d'Agents Thérapeutiques Université Paris 13 Sorbonne Paris Cité Bobigny France jolanda.spadavecchia@univ-paris13.fr; Laboratoire Micro et Nanomedecines Translationnelles, Inserm 1066, CNRS 6021, Institut de Recherche en Ingénierie de la Sante, Bâtiment IBS Institut de Biologie de la Sante, Université, Angers, Centre Hospitalier Universitaire Angers France

## Abstract

This study promotes an innovative synthesis of a nanotheragnostic scaffold capable of targeting and destroying pancreatic cancer cells (PDAC) using the Biotinylated NFL-TBS.40-63 peptide (BIOT-NFL), known to enter various glioblastoma cancer cells (GBM) where it specifically destroys their microtubule network. This recently proposed methodology (P7391FR00-50481 LIV) applied to other peptides VIM (Vimentin) and TAT (Twin-Arginine Translocation) (CPP peptides) has many advantages, such as targeted selective internalization and high stability under experimental conditions, modulated by steric and chemical configurations of peptides. The successful interaction of peptides on gold surfaces has been confirmed by UV-visible, dynamic light scattering (DLS), Zeta potential (ZP) and Raman spectroscopy analyses. The cellular internalization in pancreatic ductal adenocarcinoma (PDAC; MIA PACA-2) and GBM (F98) cells was monitored by transmission electron microscopy (TEM) and showed a better cellular internalization in the presence of peptides with gold nanoparticles. In this work, we also evaluated the power of these hybrid peptide-nanoparticles as photothermal agents after cancer cell internalization. These findings envisage novel perspectives for the development of high peptide-nanotheragnostics.

## Abstract 2

Innovative synthesis of a nanotheragnostic scaffold capable of targeting and destroying pancreatic cancer cells (PDAC) using the Biotinylated NFL-TBS.40-63 peptide (BIOT-NFL).

## Graphical abstract

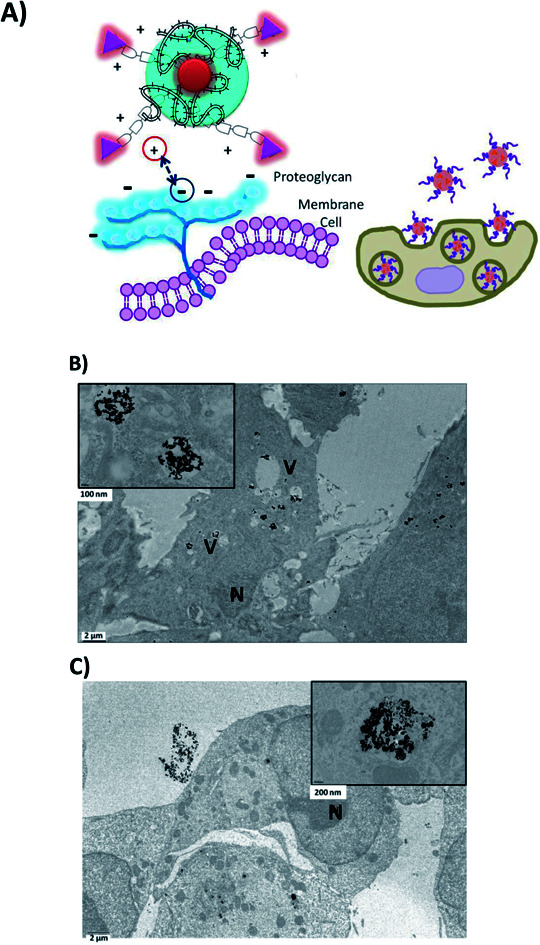

## Introduction

Gold nanoparticles (AuNPs) have singular optochemical properties, which can be due to their size, shape, and surface chemistry, and are strongly useful for several technological applications.^1–3^ These nanoparticles are also remarkable therapeutic instruments in biomedical applications, such as the delivery of drug molecules and biomarkers, due to their large surface area and their ability to selectively recognize some biomolecules such as peptides or proteins.^4–6^ Several types of peptides can be exploited to develop nanovectors that are designed to carry out a characteristic role such as active targeting of cancer cells.^7^ Cell-penetrating peptides (CPPs) are short peptides (<30 amino acids long) capable of permeating biological membranes and to enter cancer cells.^8^ In addition, specific peptide sequences are responsive to external stimuli (*e.g.*, temperature or pH) and able to check the aggregation of colloidal AuNPs. The conjugation of CPPs for specific organelle targeting is a first example in order to improve their targeted localization in nuclei or other organelles.^9,10^ NLS (nuclear localization sequence) peptides have been studied to implement this aim. Feldheim and colleagues described the delivery of bovine serum albumin (BSA) coated gold nanoparticles after internalization into HepG2 cells with a diameter of 20 nm to NLSs of different viruses.^11,12^ The conjugation of TAT (twin-arginine translocation) is another method to show a better cellular internalization and nuclear localization of nanomedicine.^13^ This method has been developed by de la Fuente *et al.*, who grafted TAT to tiopronin onto gold nanoparticles of adequate size (2.8 nm in diameter) to cross through the nuclear pores of primary human fibroblast cells (hTERT-BJ1).^14^ Conde *et al.* functionalized 14 nm gold nanoparticles with PEG chains, an arginine–glycine–aspartic (RGD) targeting peptide and TAT peptide.^15^ Previously, J. Eyer and his team discovered the sequences named ≪tubulin binding sites (TBSs)≫, located along the intermediate filaments which are able to bind free tubulin. They then revealed a peptide, NFL-TBS.40-63 ( neuro filament low subunit-tubulin binding site 40-63, also called NFL-peptide), which can enter or interact specifically in different glioblastoma cell lines (mouse, rat, human and canine).^16–18^ The NFL-peptide inhibits *in vitro* the cell proliferation and *in vivo* the tumour development.^17–19^ In the last few years J. Spadavecchia *et al.* widely studied the chemical mechanism of hybrid gold nanoparticles through complexation methodology (Method IN) and the influence of several drugs,^20–24^ biomolecules (proteins, enzymes, and biological cofactors) as capping agents and/or reagents on the growth of the nanoparticle process.^21–25^

**Scheme 1:**
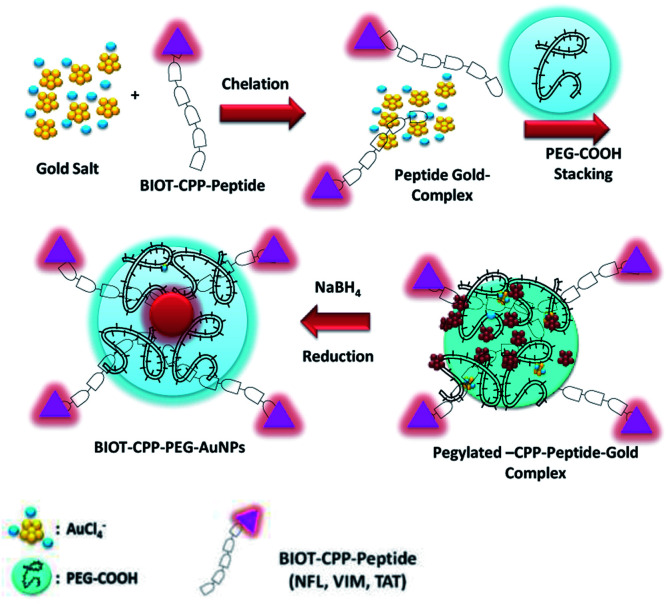
Chemical design of the synthesis of BIOT-CPP-PEG-AuNPs (BIOT-NFL-PEG-AuNPs; BIOT-VIM-PEG-AuNPs; BIOT-TAT-PEG-AuNPs) by the complexation methodology (Method IN).

In the present study, PEG molecules and BIOT-CPP-peptides (BIOT-NFL; BIOT-VIM; or BIOT-TAT-peptides) both participate in the stabilization of AuNPs *via* complexation between their ketone and amino groups with chloride auric ions. In our case, the formation of AuNPs from AuCl_4_^−^ comprehends the subsequent steps (Scheme 1):

(1) Chelation of biotinylated CPP peptides (NFL, VIM, or TAT) with AuCl_4_^−^ to generate a hybrid peptide gold cluster.^22^

(2) The embedding process of the PEG diacid polymer onto the hybrid peptide gold clusters.

(3) Final reduction and colloidal stabilization of hybrid peptide gold nanoparticles (BIOT-CPP-PEG-AuNPs).

In the first step, each biotinylated peptide (NFL; VIM; or TAT) was mixed in an aqueous gold salt solution (HAuCl_4_), to complex with it through the positively amino charged (–NH_3_^+^ and –NRH_2_^+^) water solution. The presence of the biotin onto the peptide confers a better chemical configuration to govern specific targeting.^26,27^

The addition of PEG diacid to the biotinylated peptide–gold complex (BIOT-CPP-AuCl_4_^−^) promotes the kinetics of reduction by chelation with the Au ions.^28^

Based on these findings, some authors have recently conceived and protected the role of biotinylatedCPP peptides (NFL, VIM, and TAT) as hybrid theragnostic complexes (P7391FR00-50481 LIV). In the present study, the aim was to describe the chemical design and development of these novel nano theragnostic molecules by using biotinylated-CPP (BIOT-CPP) complexed to gold ions to obtain highly stable nanoparticles (BIOT-CPP-PEG-AuNPs). Then, we characterized all colloidal systems by spectroscopic techniques, including UV-visible spectroscopy, dynamic light scattering (DLS) analysis, zeta potential analysis, Raman spectroscopy and transmission electron microscopy (TEM) to validate the success of the chemical protocol. The obtained results demonstrate that this approach provides particularly stable nanoparticles (up to one year).

Then the interaction of our hybrid nanoparticle systems with pancreatic (PDAC; MIA PACA-2) and glioblastoma (GBM; F98) cancer cells was observed to evaluate their cellular internalization.

Our results confirm the excellent properties of these hybrid nanoparticles as a theragnostic agent. Moreover, this biological activity is also very stable (up to one year).

All these results show a new method for producing hybrid nanoparticle platforms which reveal to be particularly effective for cellular internalization and which are extremely stable. Based on these observations, we have the prospect of a new way to apply our nanoformulations as smart chemotherapeutic agents in the field of drug delivery to simultaneously treat primary cancer (*i.e.* pancreatic cancer) and possibly localized lung and brain metastases.

Our work predicts a way for the development of similar innovative theranostic platforms, allowing the detection of protein-associated tumors and the simultaneous cancer treatment boosting the immune system and suppressing the angiogenic phenomenon.

## Results and discussion

### Physico-chemical evaluation of BIOT-NFL-PEG-AuNPs

Previously, J. Eyer *et al.* conducted several studies concerning the biological activity of intermediate filaments to fix tubulin dimers on specific sites named tubulin-binding sites (TBS).^29^ It was demonstrated that several peptides corresponding to these sequences can affect tubulin polymerization,^17^ and some (*e.g.* VIM-TBS) can penetrate into nuclei of glioma cells.^39^ A peptide corresponding to the TBS sequence located on the Neurofilament light subunit (NFL-TBS.40-63) (Fig. S1 in ESI†) can interact *in vitro* with tubulin and can enter multiple glioma cell lines where it inhibits the proliferation of glioma cells by altering their microtubule network. Moreover, when lipid nanocapsules (LNCs) are functionalized with the NFL-TBS.40-63 peptide, it is possible to target *in vitro* and *in vivo* their entry into glioma cells.^16,17^ To further evaluate the capacity of cell internalization, several chemical methods have been tested to graft the peptide (NFL-TBS.40-63) through a biotin- or amino-polymer modified onto a LNC, but without success for many of them, probably due to an alteration of the structure and/or biological activity of the peptide during the chemical grafting (data not shown). On the contrary, electrostatic incubation of the LNC with the NFL-TBS.40-63 peptide allowed an increased cellular uptake.^16–18^ In the present study, we proved that our methodology of complexation (Method IN)^20^ to conjugate the BIOT-NFL-peptide on gold surfaces preserved all optochemical characteristics and stability under experimental conditions, increasing the power of internalization in both PDAC and GBM cells.

**Fig. 1:**
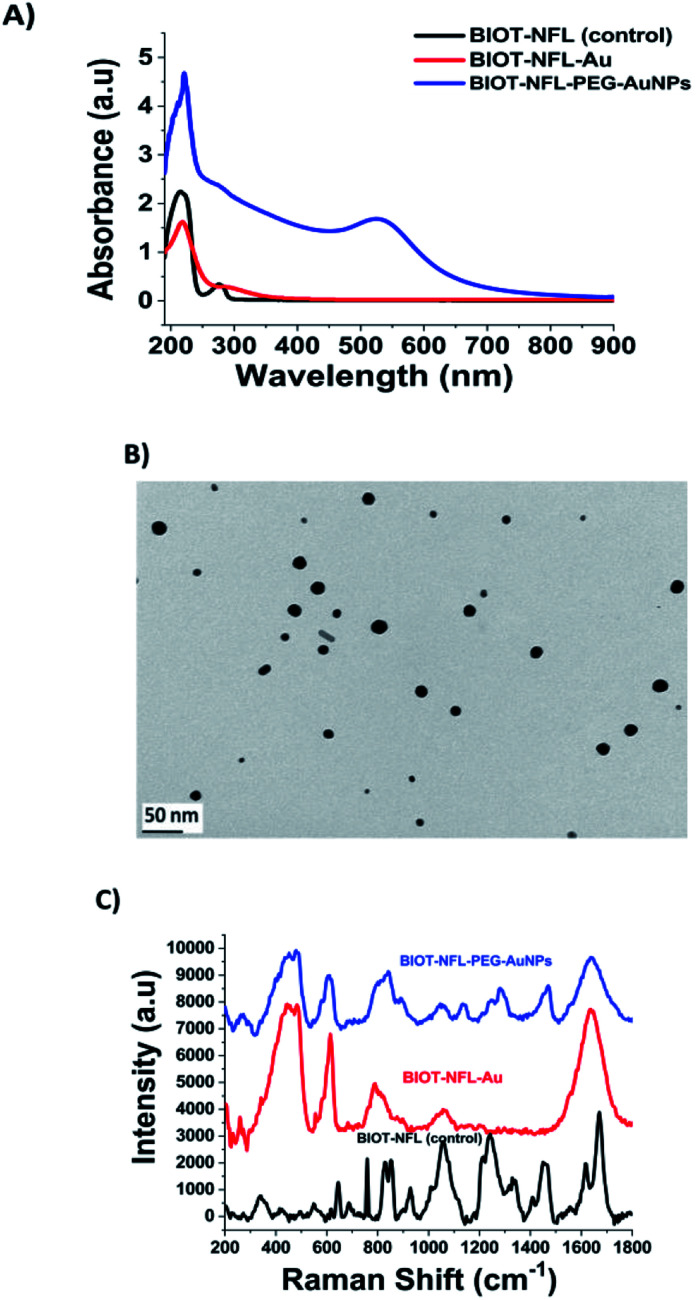
(A) Normalized UV-Visible absorption spectrum of BIOT-NFL-PEG-AuNPs. (B) TEM images of BIOT-NFL-PEG-AuNPs. Scale bar: 50 nm. (C) Raman spectrum of BIOT-NFL-PEG-AuNPs (blue line) compared to pegylated gold complex BIOT-NFL-Au (red line) and the free BIOT-NFL-peptide spectrum as controls (black line). Experimental conditions: *λ*_exc_ = 785 nm; laser power 20 mW; accumulation time: 180 seconds.

As illustrated in Fig. 1-A, the black line and the UV-Vis spectra of the BIOT-NFL-peptide display a small peak at 275 nm and a characteristic peak at 215 nm due to the π–π* electronic transitions related to peptide's backbone. After complexation of BIOT-NFL-AuCl_2_^−^ (first step), we observed a disappearance of the peak at 275 nm and a decrease of the peak at 218 nm (Fig. 1A, red line). This spectroscopic characteristic was associated with π–π* electronic transitions due to interactions between the NFL-peptide backbone and gold salt ions and gives clear evidence of the complex formation, with a variation of the steric arrangement of the peptide in the gold–salt complex.

In the second step, PEG diacid is added to the BIOT-NFL-peptide-AuCl_2_^−^ dispersion, leading to the formation of BIOT-NFL-AuCl_2_-PEG diacid complexes as previously described^22^ (spectra not shown). One possibility is that, during the second step, Au(iii)-BIOT-NFL complexes migrate through PEG molecules as described previously with other biomolecules.^20,21^ Lastly, the reduction mixture with NaBH_4_ was obtained in order to reduce the gold species from Au^III^ to Au^0^ and form the final product BIOT-NFL-PEG-AuNPs (Fig. 1A, blue line). The reduction of Au proceeds *via* electron transfer at the surface of electron-rich, BIOT-NFL-AuCl_2_-PEG diacid molecule capped, polydisperse gold particles with a positive charge (+24.7 mV) as confirmed by dynamic light scattering (DLS) and zeta potential analyses (Table 1). The absorption spectra of BIOT-NFL-PEG-AuNPs were characterized by a prominent surface plasmon band at 525 nm (Fig. 1A, blue line). The TEM images of BIOT-NFL-PEG-AuNPs display polydisperse nanoparticles with an average size of 19 nm obtained by using the ImageJ software (Fig. 1B). This polydispersity is due to various steric orientations of BIOT-NFL-peptide on the crystallographic gold facet. The hydrodynamic diameter measured by the DLS technique is about 91 ± 2 nm due to steric arrangement of the polymer in the gold peptide complex. Other authors have realized that the synthesis of analogous nanostructures added macromolecules and biopolymer molecules to the growth dispersion of AuNPs.^28^ Based on previous results,^20–22^ we suggest that when BIOT-NFL-peptide and dicarboxylic PEG were mixed with the AuCl_4_^−^ dispersion, the PEG was bound initially to Au(iii) in a mushroom conformation.^23^ The presence of biotin onto the peptide improves the chemical and steric configurations and consequently active targeting on cancer cells.

**Table 1:** Zeta potential and the hydrodynamic diameter of BIOT-CPP-PEG-AuNPs

CPP nanoparticles	Zeta potential (mV)	Hydrodynamic diameter (nm)	PdI
BIOT-NFL-PEG-AuNPs	+24 ± 0.7	91 ± 2	0.25
BIOT-VIM-PEG-AuNPs	−33 ± 0.8	101 ± 2	0.16
BIOT-TAT-PEG-AuNPs	−29 ± 0.6	104 ± 2	0.18
PEG-AuNPs	−24 ± 0.2	25 ± 2	0.38

In addition, the bright pink-violet color of the colloidal solution and the UV-Vis spectra remain unaltered after storage for more than 12 months at room temperature, suggesting the formation of a stable particle suspension.

The steric arrangement of BIOT-NFL-peptide and pegylated gold nanoparticles was confirmed by Raman spectroscopic analysis (Fig. 1C). In detail, the Raman spectra of each step of synthesis exhibit many bands in the region 200–2000 cm^−1^. Considering the curve of free BIOT-NFL-peptide as a control (Fig. 1C, black line), the spectra are dominated by the characteristic Raman signals caused by the tyrosine residue vibrations.^32,33^ An intensity ratio of a Tyr doublet was observed at 827–852 cm^−1^ (BIOT-NFL-peptide contains 2 Tyr residues in its primary structure), due to a resonance between the ring-breathing vibrations and a para-substituted benzene ring of Tyr,^33,34^ determined by the nature of the hydrogen-bonding of the phenolic hydroxyl in the Tyr residue.^34^ The bands observed in the spectral range of 990–890 cm^−1^ are attributed to the contribution of the aliphatic side-chain vibrations of Asp, Asn, Glu, Gln, Lys, and Arg. The NFL-peptide contains thirteen of these residues in the amino acid sequence.^35,36^ The presence of His is also manifested by the very weak peak at 1554 cm^−1^. There are some other bands, which could be assigned to the aliphatic side-chain modes, especially of Arg, at 1460 [d(CH_2_)], 1446 [d(CH_2_) and/or (NH)], 1342 [(CH_2_)], 1174 [(NH_2_)/(NH_3_^+^)], 1126–1155 [(NH_2_)] and/or (CCN)], 1105 (CCN) and/or (NH_2_)], and 1055–1051 [Arg [(NH_3_^+^) and/or (CN)]. As can be seen, the BIOT-NFL-peptide dissolved in water exhibits a peak at 1449 cm^−1^ characteristic of the biotin CH_2_-ring^37^ and amide I and III bands at 1652 and 1267 cm^−1^, respectively.^38^ The 1652 cm^−1^ Raman signal was interpreted to represent the α-helical or unordered structure, whereas the amide II mode was allocated to the β-turn structure. The strongest signal at 1652 cm^−1^ is due to the α-helical conformation.

The Raman fingerprint of the first step (BIOT-NFL-AuCl_2_^−1^; Fig. 1C, red line) product was the presence of a peak around 260 cm^−1^ and 460 cm^−1^ due to complexation between the BIOT-NFL-peptide and gold salt. Indeed, this band can be assigned to the gold chloride stretches and δ (O–Au–O). An improvement of the peak at 610 cm^−1^ is due to a tyrosine backbone that confirms a different steric arrangement of the peptide upon gold salt complexation. These chemical and steric behaviours are also confirmed by the disapparition of a double peak at 827–852 cm^−1^ and all peaks in the spectral range 1200–1400 cm^−1^.

After complexation with gold ions, stacking with PEG-diacid molecules and reduction with NaBH_4_, the Raman fingerprint of the BIOT-PEG-AuNPs (Fig. 1C, blue line) represents the presence of a double peak at 260 cm^−1^. These bands can be assigned to the gold chloride stretches, *ν* (Au–Cl), and *δ* (O–Au–O) and is clear evidence of the formation of a complex between AuCl_2_^−^ and the BIOT-NFL-peptide in solution. The common peak at 342 cm^−1^ is due to the vibrations (OH⋯O) and *ν* (OH⋯O) of the PEG. Therefore, we assume that NaBH_4_ reduced Au^3+^ to Au^0^ to form dispersed AuNPs in which BIOT-NFL was embedded between PEG-diacid chains and AuNPs. We assume that this behaviour is due to steric arrangement of the BIOT-NFL–Au complex in PEG diacid molecules. These phenomena influence the peptide-surface orientation with the change of electronic distribution within the BIOT-NFL, Au^3+^ and PEG chains during the synthetic process with formation of a new drug-gold nanoparticle system. The fingerprint of PEG-COOH on the AuNP surface was proved through the observation of the Raman bands at 1137, 1270, and 1455 cm^−1^ due to the vibrations of C–O–H, C–O–C and C–O chemical groups, as previously described.^20^ The strong broad 1659 cm^−1^ and medium relative intensity 1267 cm^−1^ SERS signals are due to the amide modes. The gap in the spectral range between 1415 and 1370 cm^−1^, where the carbonyl vibrations are expected to appear, suggests that the 1266, 1178, 1150, 1108, and 1051 cm^−1^ spectral features are due to the Arg residue oscillations rather than the N-terminal-NH_2_ modes.

It has already been described that most of the Raman bands of molecules can be significantly enhanced by their proximity to the surface AuNPs. Moreover, we also assume that the BIOT-NFL/Au interaction is still the same after the NP formation and that the BIOT-NFL is grafted at the NP surface through the complex formation with Au. The steric orientation of the peptide on the AuNP surface will be influenced by electrostatic interactions between amino groups and phenol in the presence of diacid PEG molecules under specific conditions of pH and ionic strength.

The wide band observed around 1600 cm^−1^ in the Raman spectra is assigned to water and allocated to the C=N bond stretches. Thus, based on the above observation, it can be proposed that the amide bond closest to the C-terminal Tyr interacts with the gold surface of nanoparticles, whereas the Arg residue only assists in the peptide interaction with this surface.

### Chemical and physical evaluation of BIOT-VIM-PEG-AuNPs and BIOT-TAT-PEG-AuNPs

In this study we also compared the optochemical properties of NFL-TBS.40-63 grafted on gold complex nanoparticles, with other CPP and TBS peptides such as Vim-TBS.58-81 derived from the intermediate filament vimentin and TAT-48-60 which were described previously for their aminoacid structures and mechanism of action.^39^ Unlike the synthesis of BIOT-NFL-PEG-AuNPs, the chemical approach to synthesize BIOT-VIM-PEG-AuNPs and BIOT-TAT-PEG-AuNPs has provided a double layer of PEG diacid during the stacking process with a half amount of peptides (BIOT-VIM; or BIOT-TAT) to ensure better stability.

**Fig. 2:**
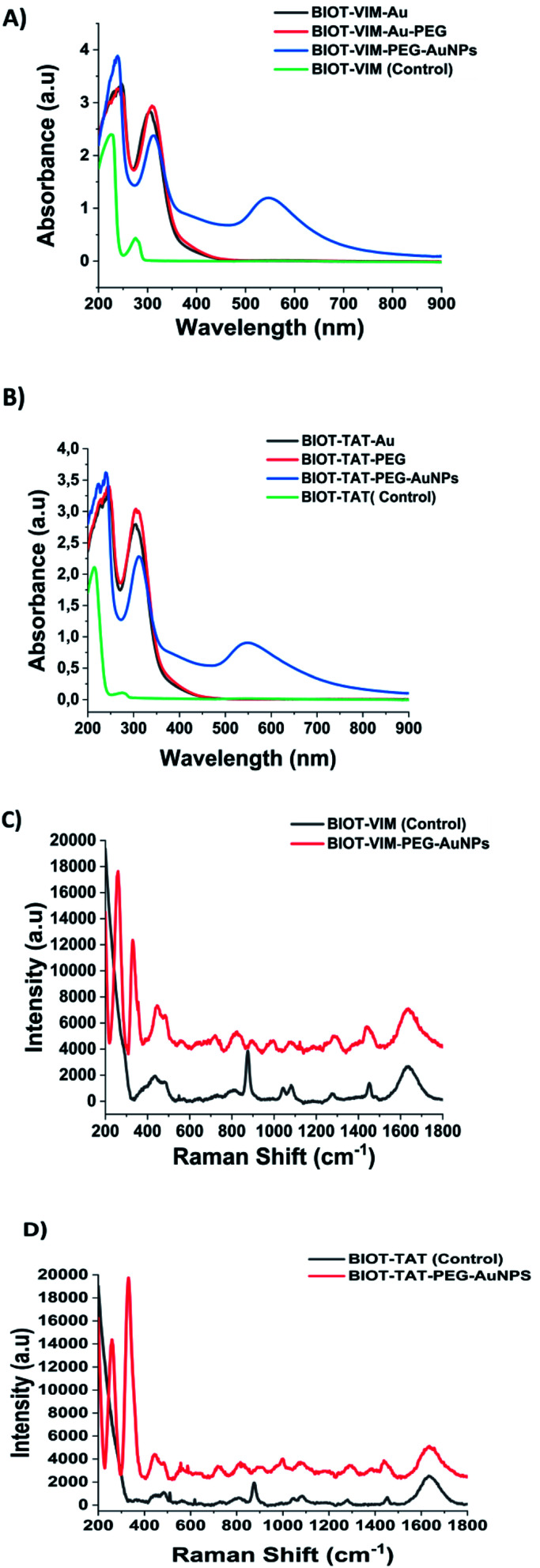
(A, B) UV-Visible absorption spectra in the range 200–900 nm of BIOT-VIM-PEG-AuNPs and BIOT-TAT-PEG-AuNPs (blue line) compared to those of the peptide gold complex precursor (red line) and peptide as controls (green line). (C, D) Raman spectra of BIOT-VIM-PEG-AuNPs and BIOT-TAT-PEG-AuNPs (red line) compared to those of BIOT-VIM and BIOT-TAT as controls (grey line). Experimental conditions: *λ*_exc_ = 785 nm; laser power 20 mW; accumulation time: 180 seconds.

The BIOT-VIM-peptide showe characteristic UV-Vis absorption spectra at 277 nm and 214 nm due to electronic π–π* delocalization of the BIOT-VIM peptide (Fig. 2A, green line). When BIOT-VIM-peptide was added to the gold salt mixture, we observed a double absorbance peak at 243 and 304 nm due to electronic transitions between BIOT-VIM and AuCl_4_^−^, confirming the complexation (Fig. 2A, green line). The stacking step by using the polymer (PEG-diacid) in the mixture reaction does not influence the spectroscopic fingerprint of the peptide gold complex (Fig. 2A, red line), contrary to previously published papers in which the presence of the PEG-diacid in the mixture reaction influenced the formation of a metallo-micelle.^20^ After reduction with NaBH_4_, the apparition of the plasmon peak at 546 nm confirms the formation of the hybrid gold nanoparticles (Fig. 2A, blue line). The reduction of Au proceeds *via* electron transfer at the surface of electron-rich BIOT-VIM-AuCl_2_-PEG molecule capped gold particles. The absorption spectra of BIOT-VIM-PEG-AuNPs were characterized by a small red shift at 312 nm and a surface plasmon band at 546 nm (Fig. 2A, blue line). A comparable spectroscopic behaviour was confirmed for BIOT-TAT-peptide (Fig. 2B, green line), for which a similar UV-Vis absorption spectrum was observed. The presence of a double layer of pegylated chains on the peptide gold complex (BIOT-VIM-AuCl_2_; BIOT-TAT-AuCl_2_) was confirmed through the presence of a negative charge characterized by zeta potential and dynamic light scattering (DLS) analyses (Table 1).

Comparing the Raman spectra of both peptides (BIOT-VIM and BIOT-TAT) before (black line) and after nanoparticle formation (BIOT-VIM-PEG-AuNPs; BIOT-TAT-PEG-AuNPs) (red line) (Fig. 2C and D), we observe in both cases characteristic tyrosine residues vibrations at 438–481 cm^−1^, *ν* (C–C), Tyr breathing at 879 cm^−1^, 821 cm^−1^, and 890 cm^−1^, and arginine vibrations (*δ* [NH_2_] *ν* (C–N) and/or *δ* (CH_2_)). The peak at 1440 cm^−1^ in both peptides confirms the presence of biotin well distributed on the gold surface of nanoparticles after colloidal formation. The Raman fingerprint of PEG-diacid was also confirmed and discussed previously.^20^ The success of peptide-gold complexation in both cases (BIOT-VIM and BIOT-TAT) was confirmed by a double peak at 258 and 328 cm^−1^. These bands can be assigned to the gold chloride stretches, *ν* (Au–Cl), and *δ* (O–Au–O), and it is clear evidence of the complex formation between AuCl_2_^−^ and BIOT-VIM/TAT. In the case of BIOT-TAT, the peak at 329 cm^−1^ is more prominent than that of BIOT-VIM, probably due to a different steric arrangement of the peptide. The loading of CPP-peptides (BIOT-NFL, BIOT-VIM, or BIOT-TAT) on the gold nanoparticles was carried out by centrifugation under specific conditions and monitored by UV-Vis spectroscopy. We observe the presence of the peaks at 234 nm and 312 nm due to gold salt and pegylated chains in excess, and the disappearance of the peak at 275 nm is characteristic of the peptide. This result confirms that the peptide was grafted entirely on the gold nanoparticles during the growth process.

### Stability of biotinylated-CPP-PEG-AuNPs (BIOT-NFL, BIOT-VIM, and BIOT-TAT)

As described in the literature, CPPs are small biomolecules with a high amphiphilic character that shows a poor bioavailability with a tendency to aggregate after interaction with the solvents and membranes.^40,41^ Peptide stability in aqueous solutions is influenced by several chemical and physical parameters.^42^ Some non enzymatic degradation processes include chemical reactions^43^ such as acid/base hydrolysis,^44^ deamidation,^45^ oxidation,^46^ and disulfide exchange with implications of some amino acids (*i.e.*, asparagine, tyrosine, methionine, and histidine).^47^ For this reason, they are considered as poor drug candidates with several limitations for therapeutic applications. Recently, pharmaceutical companies have rediscovered a scientific interest to invest economically in order to surpass these limitations. For this purpose, there are several chemical synthetic strategies to improve peptide stability and productivity.^48^ These parameters include stereo chemical orientation,^49^ different conformation and polymer conjugation.^50^

**Fig. 3:**
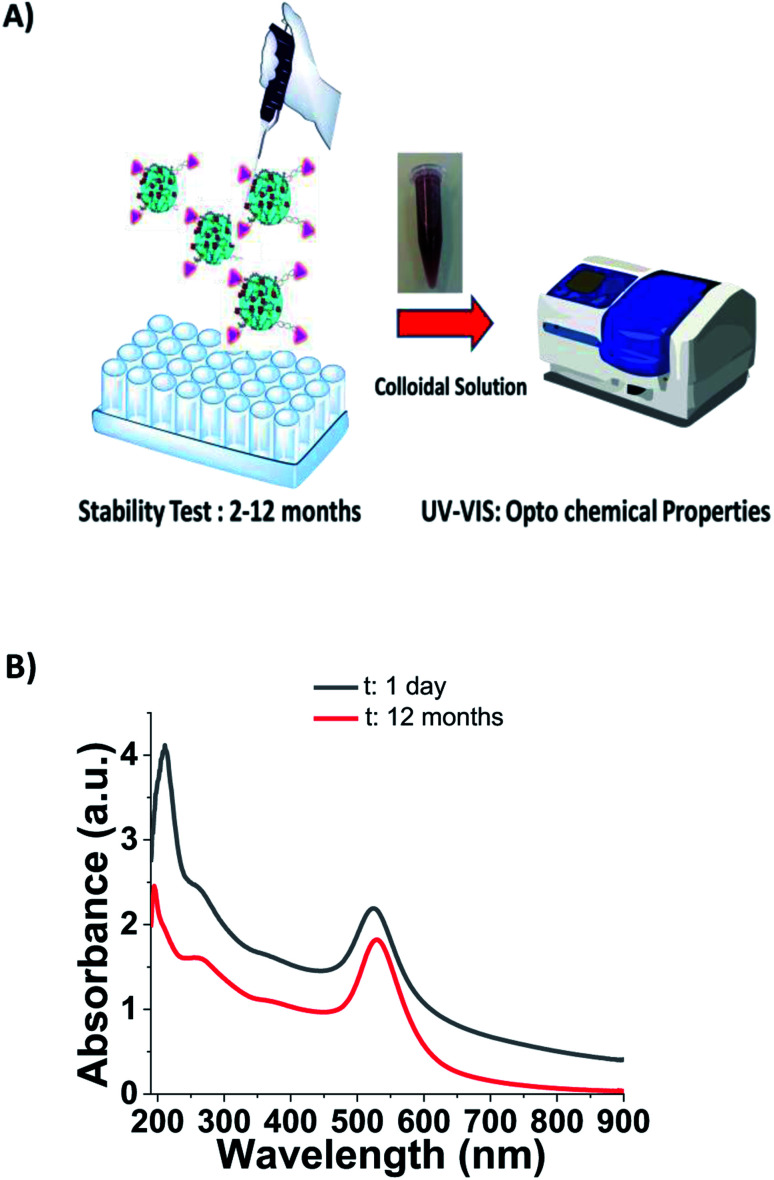
Schematic representation of the stability test during 2–12 months of biotinylated-CPP-colloidal solution under experimental conditions and subsequent spectroscopic characterization (A). UV-visible spectra of BIOT-NFL-PEG-AuNPs under experimental conditions a t: 1 day (black line) and after 12 months (red line) (B). (Please note that the drawings are not in scale and are not intended to be representative of the full sample composition).

To evaluate the stability of BIOT-CPP-PEG-AuNPs (NFL, VIM, or TAT-peptides) as prospective theragnostic delivery tools, all colloidal solutions were stored at room temperature and monitored every time *via* the absorption spectra to check all optochemical properties (Fig. 3A). Localized surface plasmon (LSP) band analysis was performed in water dispersion at room temperature and under the shelter of light over a reasonable period of time (from 2 to 12 months). As in the first case, the synthesized BIOT-NFL-PEG-AuNPs did show any change in the LSP band position (525 nm) over a period of twelve months in water solution at the pH 6 (Fig. 3B).

**Scheme 2:**
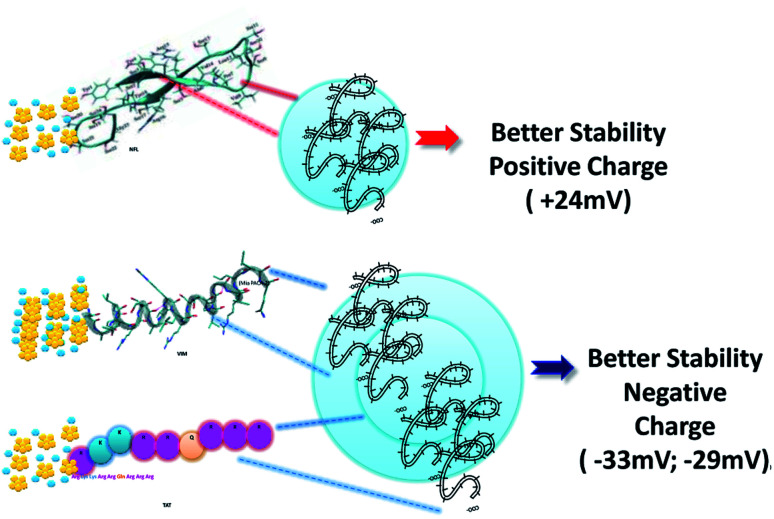
Schematic representation of pegylated stacking process (NFL and VIM structures were made based on the literature).

As described previously, PEGylated nanoparticles functionalized with 16.8 and 25.2 μg mL^−1^ of PEG remain stable under experimental conditions.^23–25^ In our case, BIOT-NFL-PEG-AuNPs preserves its morphology and size for 1 year, which is a remarkable result to allow pharmaceutical applications. The analogous behavior was confirmed for BIOT-VIM-PEG-AuNPs and BIOT-TAT-PEG-AuNPs that showed a similar stability test result of about 2 months (Fig. S2A, B in ESI†). In the last few years, some authors have studied the high stability effect of CPP-peptides after conjugation with PEGylated chains at a specific length (PEG 1000) and/or after inclusion into polymeric-pegylated micelles with PEG 5000-PE.^51–53^ The same team further established a direct correlation between the PEG length used for stacking and proteolytic protection. In our methodology, we used a symmetric short pegylated polymer chain with two carboxylic groups on the opposite end that play an important role during the complexation and stacking.^20^ It was proved that any polypeptide chain assumes a specific native conformation responsible for their stability. Therefore, intra or intermolecular interactions allow a stable folded conformation that positively contributes to peptide stabilization and decreases peptide degradation and metabolization. Based on these findings, we can assume that electrostatic embedding between PEG-diacid and gold-complex-biotinylated peptide (BIOT-CPP-AuCl_2_) increases the stability of each peptide. As showed in Table 1, BIOT-NFL-PEG-AuNPs shows a positive charge contrary to BIOT-VIM/TAT-PEG-AuNPs. This chemical behaviour is due to several chemical and steric conformations of each peptide with PEGylated chains and their electrostatic interaction between amino acids and carboxylic groups (Scheme 2).

In the case of BIOT-NFL-PEG-AuNPs, the NFL-peptide was added at an accurate concentration (1 mg mL^−1^) that allows a better electrostatic interaction during polymeric stacking that favours a better stability with a positive charge of about +24 ± 0.7 mV. Contrary to BIOT-VIM/TAT-PEG-AuNPs, VIM and TAT-peptides were added at a concentration of 0.07 mg mL^−1^ to maintain a moderate chemical stability with a double layer of pegylated chains responsible for a negative charge as shown in Table 1. Despite the double layer of PEG in the synthetic process of BIOT-VIM/TAT-PEG-AuNPs, we can assume that BIOT-NFL-PEG-AuNPs, due to the specific conformation of the peptide in the nanoparticle, is the best candidate in terms of stability and for further pharmaceutical applications.

### Cancer cell internalization: hypothesis and the mechanism

Several chemical and physical investigations have been realized to analyze the cellular internalization of NPs.^6–8^ For example, targeted groups can enhance the absorption process, and stimulus groups can improve the release tolerance of nano transporters as drug delivery systems.^54^ Several studies have proved that the plasmonic characteristics of NPs, such as the size,^55,56^ shape, and surface morphology,^57^ significantly influence their cellular internalization. Cell penetrating peptides (CPPs) can enter into cells by energy-dependent or independent mechanisms.^58^ The peptide-membrane interactions play a key role during cell interaction and penetration.^59^ The three major parameters for the CPP internalization into cellular membranes are the peptide concentration, their sequence, and lipid composition of the membrane.^58,59^

Concerning the peptide concentration, the molecular mechanism for the uptake of some cationic CPPs is different. When the concentration is increased, rapid cytosolic uptake is revealed, and when the concentration of the peptide is low, the mechanism of uptake prevails.^58^ The second crucial parameter is the peptide sequence as previously described for TAT-peptide rich in arginine and positively charged.^8–58^

Three possible mechanisms were established for the internalization of CPPs: (i) the first is direct penetration, which is an energy-independent pathway; (ii) the second mechanism is the endocytosis pathway, energy dependent; (iii) the third mechanism is translocation through the formation of a transitory structure. In this study, the interaction of the CPP takes place with the cellular membrane, which improves the disruption of the membrane lipid bilayer.^60^

**Fig. 4:**
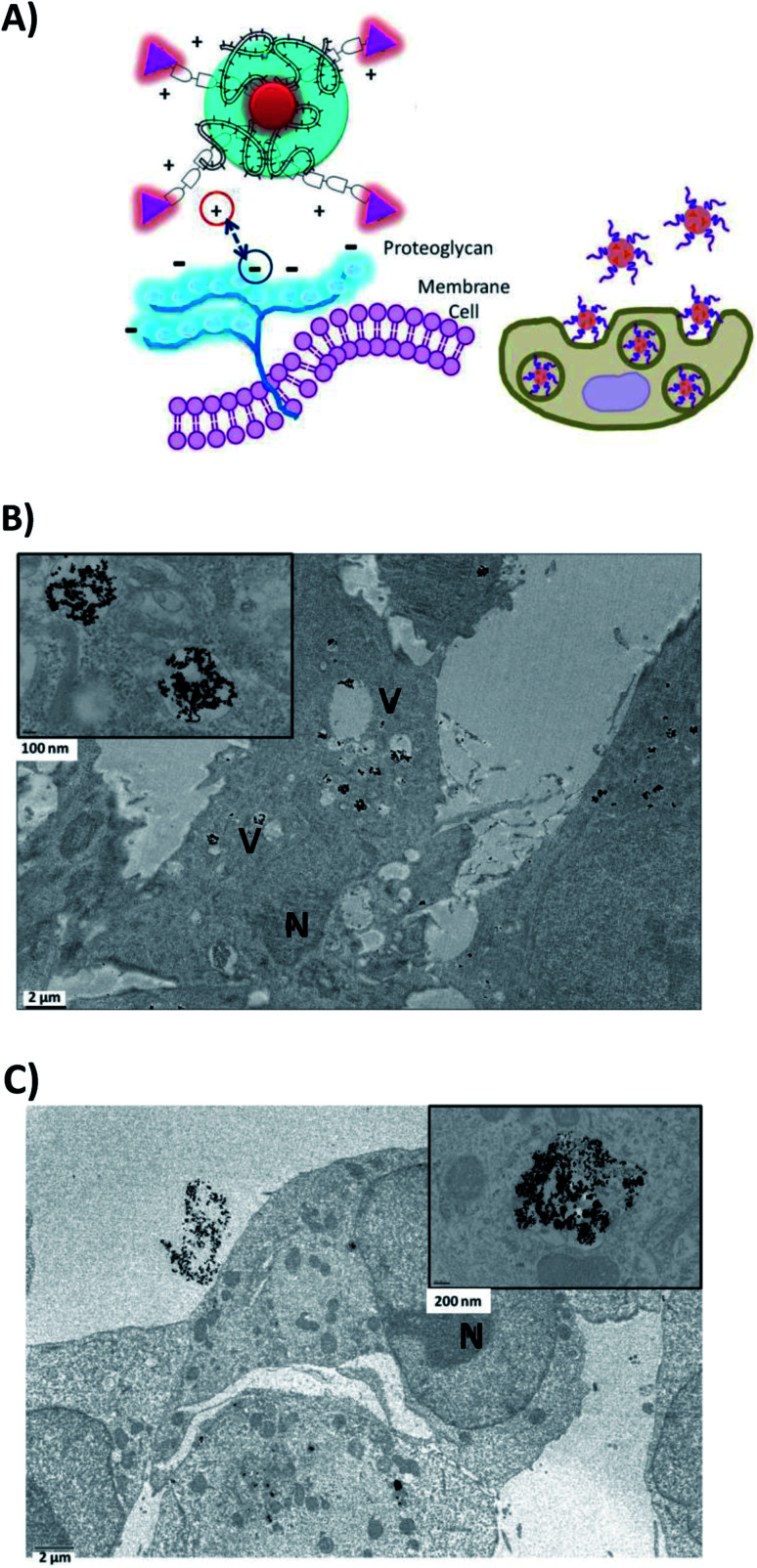
(A) Schematic mechanism of internalization of BIOT-NFL-PEG-AuNPs (B) in F98 cancer cells or (C) in MIA PACA-2 cancer cells.

In this paper, we evaluated the capacity of PEG-AuNPs combined or not with the BIOT-CPP-peptides to be internalized in PDAC (MIA PACA-2) and GBM (F98) cells (Fig. S3, ESI;† cell controls (NT)). All cells were internalized with PEG-AuNPs alone at 500 μmol L^−1^ as a control (Fig. S4A and S4B in the ESI†) and with BIOT-NFL-PEG-AuNPs (Fig. 4A) internalized for 72 hours (Fig. 4B and C).

**Fig. 5:**
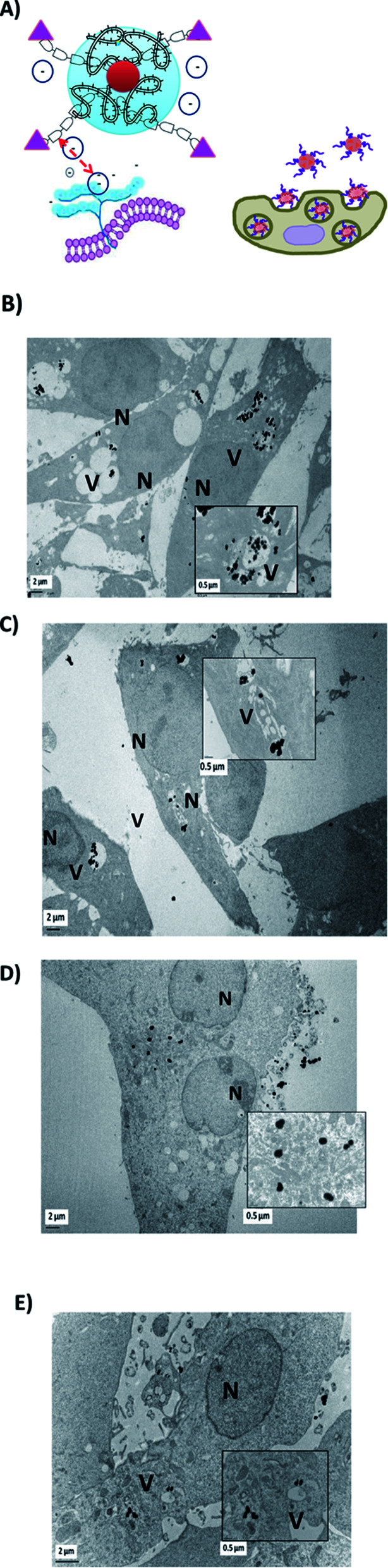
(A) Schematic mechanism of internalization of BIOT-TAT/VIM PEG-AuNPs. Electron micrographs of F98 cancer cells treated with BIOT-TAT-PEG-AuNPs (B) and with BIOT-VIM-PEG-AuNPs (C); electron micrographs of MIA PACA-2 cancer cells treated with BIOT-TAT-PEG-AuNPs (D) and BIOT-VIM-PEG-AuNPs (E).

Cells were also internalized with PEG-AuNPs alone at 250 μmol L^−1^ with BIOT-TAT and with BIOT-VIM-PEG-AuNPs for 24 hours. Then, all cells were fixed, included, and sectioned with an ultramicrotome and characterized by transmission electron microscopy. The first step during the internalization process is the electrostatic interaction between the nano-peptide and the cell surface proteoglycans, which influences the lipid supramolecular organization (Fig. 5A). This interaction may involve changes in the membrane invagination.^26^ The consequently hydrophilic environment enables accumulation of the peptide, destabilization of the micelle and release of the peptide–cargo complex in the cytoplasm. Our results showed that non treated F98 cells display a normal nucleus and cytoplasm. When cells were treated with PEG-AuNPs alone, few gold nanoparticles are present in the cells and mostly in cellular vacuoles (Fig. 4). After cellular treatment with BIOT-NFL-PEG-AuNPs, more gold nanoparticles are detected in cells, which also showed more vacuoles (Fig. 5B and C). The addition of the BIOT-NFL-peptide to cells altered their morphology and inhibited their cellular extensions. These results indicate that the presence of the BIOT-NFL-peptide increases cellular internalization in both cell lines: MIA PACA-2 in Fig. 4B and F98 in Fig. 4C. Following the treatment with BIOT-TAT-PEG-AuNPs and BIOT-VIM-PEG-AuNPs, more particles were observed in the cells; more vacuoles are present in the cells, and we observed few cells (Fig. 5B–E). A major difference was observed with BIOT-TAT and BIOT-VIM-PEG-AuNPs, in which the morphology of particles is different, which is like snow-shape. This conformational difference is probably due to the negative charge of pegylated double layer and different concentrations of BIOT-TAT and BIOT-VIM-PEG-AuNPs than that of BIOT-NFL-PEG-AuNPs that influence their interaction with the lipid membrane with consequently variation of shape.

### Photothermal activity

**Fig. 6:**
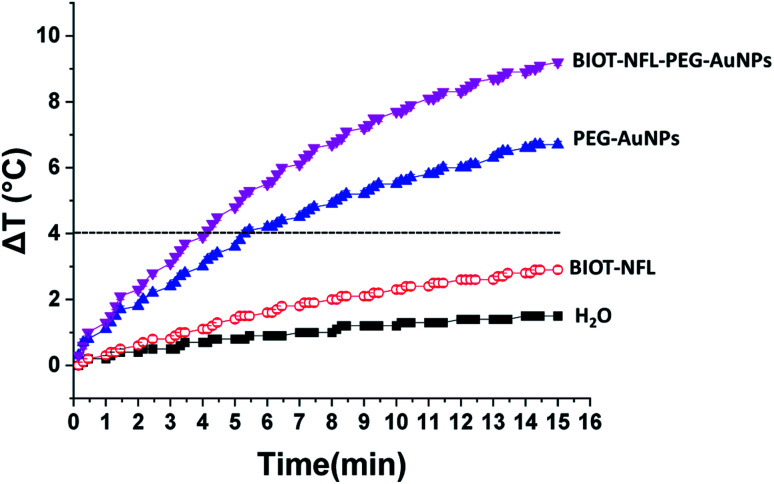
Time-dependent temperature elevation of BIOT-NFL-PEG-AuNPs, BIOT-NFL-peptide, PEG-AuNPs and control solutions under 808 nm laser irradiation (0.5 W cm^−2^). The temperature elevation of AuNPs is higher than the minimum temperature required for PTT (+4 °C).

**Fig. 7:**
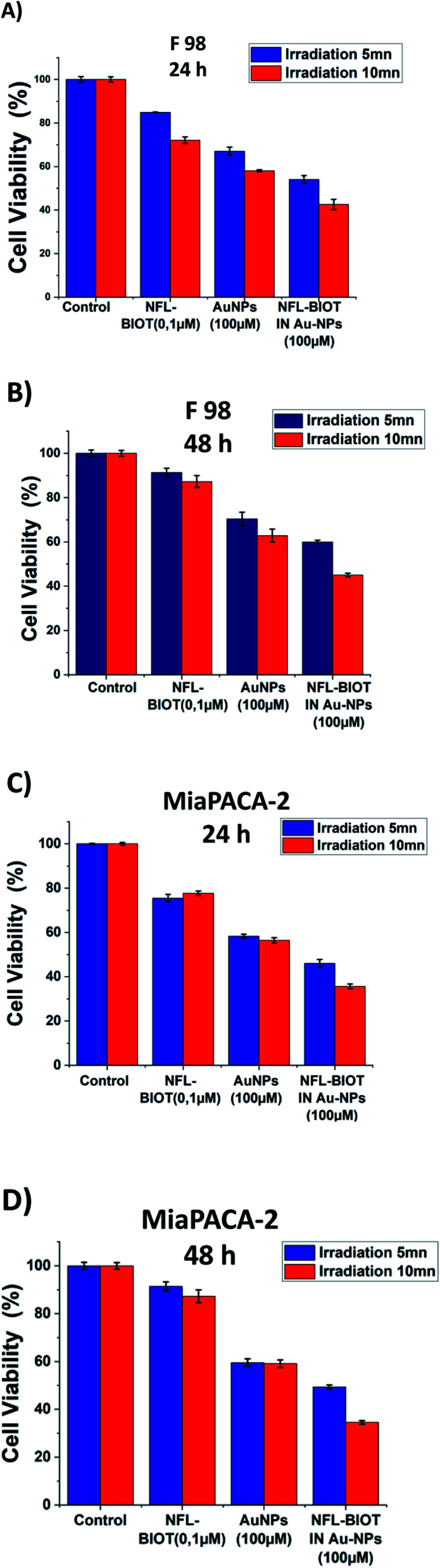
Viability test on MIAPACA-2 cells, after irradiation and internalization of nanoparticles for 24 hours (A) and after 48 hours (B). Viability test on F98 cells, after irradiation and internalization of nanoparticles for 24 hours (C) and after 48 hours (D).

Fig. 6 shows the temperature elevation characteristics in the different dispersions with and without BIOT-NFL-PEG-AuNPs *versus* time, with concentrations of BIOT-NFL (100 μmol L^−1^), and PEG-AuNPs as controls. The different dispersions were exposed to 0.5 W cm^2^ excitation laser density for 15 minutes at 808 nm wavelength to evaluate their temperature elevation in the perspective of photothermal therapy application. During laser heating of BIOT-NFL-PEG-AuNPs suspended in water dispersion (H_2_O), the near-infrared light absorbed by the AuNPs is converted to thermal energy that leads to an increase of the nanoparticle temperature and hence the surrounding medium (H_2_O). As shown in Fig. 7, the temperature of the control dispersions (H_2_O, without AuNPs) is very weak (<0.4 °C) due to the absence of the thermoplasmonic effect, while the temperature elevations of the four AuNP dispersions are much higher than the limit of 4 °C (minimum temperature required for the photothermal effect).^61^ The temperature elevation in the BIOT-NFL-PEG-AuNP dispersion is higher than that in the PEG-AuNP dispersion. MTT tests were performed to choose the optimal concentrations for photothermal tests. Indeed, we could see that at these concentrations (100 μmol L^−1^), the nanoparticles had no therapeutic effect, but after the photothermal treatment, we can clearly see their effect on the cells. We can see that the irradiation treatment has no effect on the cells alone without nanoparticles (control). In the presence of the peptide, a similar effect with or without irradiation was observed. About the PEG-AuNPs without peptide, we observed a decrease into cell viability of 60% at 24 and 48 hours of internalization (Fig. 7). For BIOT-NFL-PEG-AuNP nanoparticles, we can clearly observe a significant decrease in cell viability, which is around 60% for 5 minutes and 45% at 10 minutes after irradiation treatment, and after 48 hours of internalization, the cell viability is around 40% for 5 minutes of irradiation and 30% for 10 minutes of irradiation (Fig. 7).

## Experimental

### Materials and methods

Tetrachloroauric acid trihydrate (HAuCl_4_·3H_2_O; 99%), sodium borohydride (NaBH_4_; 98%), dicarboxylic polyethylene glycol (PEG)-600 (PEG), sodium chloride (NaCl) (0.9%; 99.5%), and phosphate-buffered saline solution (PBS) were purchased from Sigma-Aldrich (Saint-Quentin-Fallavier, France). All solvents were used without any further purification. The experiments were carried out at room temperature if not specified otherwise. The biotinylated-NFL-TBS.40-63 peptide (NH_2_-YSSYSAPVSSSLSVRRSYSSSSGS-CONH_2_), also called BIOT-NFL-peptide, was synthesized by the PolyPeptide Group (Strasbourg, France). The biotinylated-vimentin-peptide (BIOT-VIM-peptide; BIOT-GGAYVTRSSAVRLRSSVPGVRLLQ-CONH_2_) and the biotinylated-TAT-peptide (BIOT-TAT-peptide; BIOT-GRKKRRQRRRPPQ-CONH_2_) were synthetized by Millegen (Toulouse, France).

### Synthesis of CPP-PEG-AuNPs

All colloids of BIOT-CPP-PEG-coated AuNPs (BIOT-NFL-PEG-AuNPs, BIOT-VIM-PEG-AuNPs, and BIOT-TAT-PEG-AuNPs) were prepared by a well assessed chemical reduction process depicted in Scheme 1 and described as below.

### Synthesis of biotinylated-NFL-TBS.40-63 peptide nanoparticles (BIOT-NFL-PEG-AuNPs)

20 mL of HAuCl_4_ aqueous dispersion (1 mM) was added to the NFL-peptide solution (0.08 mL, 1 mg mL^−1^ in water/10% ethanol) and stirred vigorously for 10 minutes. Then, 250 μL of a stabilizing agent dicarboxylic PEG (PEG-COOH) was added and mixed by magnetic stirring at room temperature. Finally, 1.2 mL of aqueous NaBH_4_ (3 mg/10 mL) was added at once. The formation of the BIOT-NFL-PEG-AuNPs was indicated by an instantaneous color change of the dispersion from pale yellow to bright pink-purple after the addition of the reducing agent. The “as-prepared” BIOT-NFL-PEG-AuNP dispersion was purified by centrifugation three times at 10 000 rpm for 10 minutes; then, the supernatant was discarded. The pellet was redispersed two times in an equivalent amount of water to remove excess of non-conjugated dicarboxylic PEG and BIOT-NFL-peptide, respectively. The colloidal dispersion was stored at 27–29 °C and characterized by UV-visible spectroscopy, transmission electron microscopy (TEM), and Raman spectroscopy.

### Synthesis of BIOT-VIM-PEG-AuNPs and BIOT-TAT-PEG-AuNPs

20 mL of HAuCl_4_ aqueous dispersion (1 mM) was added to VIM-peptide solution (0.07 mg mL^−1^, in water/10% ethanol) and to TAT-peptide solution (0.07 mg mL^−1^ in water/10% ethanol) and stirred vigorously for 10 minutes. Then, 500 μL of a stabilizing agent dicarboxylic PEG (PEG-COOH) was added at room temperature. Finally, 1.2 mL at 6 mg/10 mL of NaBH_4_ (sodium tetrahydruro borate, Sigma-Aldrich) was added to the solution until a color change from yellow to red-pink. The as-prepared colloidal solution was purified, stored, and characterized as described above.

### Synthesis of pegylated gold nanoparticles (PEG-AuNPs)

Pegylated gold nanoparticles (PEG-AuNPs) used as a control were synthesized by a chemical protocol described previously.^23^

### Physico-chemical characterization

All the measurements were performed under specific experimental conditions.^23^

### UV/Vis measurements

The absorption spectra were recorded using a double-beam Varian Cary 500 UV-Vis spectrophotometer (Agilent, France). The absorption spectra of the AuNPs were recorded in water at a concentration of 10^−4^ M in the 200–900 nm spectral range.

### Transmission electron microscopy (TEM)

PEG-AuNPs and BIOT-NFL-PEG-AuNPs were observed by transmission electron microscopy (TEM) at the Service Commund’Imageries et d'Analyses Microscopiques (SCIAM; University of Angers, France). 2 μL of each sample was deposited on copper grids (150 mesh) and stained with 2% uranyl acetate for one minute, and then each sample was dried at room temperature before observation. The examination was performed using a 120 kV Jeol JEM-1400 electron microscope (Jeol, Japan) equipped with a Gatan SC1000 ORIUS^®^ CCD camera (11 Megapixel) from the USA.

### Raman Spectroscopy

The Raman experiments have been performed on an Xplora spectrometer (Horiba Scientifics-France) as described previously.^20^

### Hyperthermia/phototherapy *in vitro* test

Before the phototherapy *in vitro* test, a preliminary evaluation of nanoparticle dispersions was performed. An aqueous dispersion of BIOT-NFL-PEG-AuNPs (1 mL), as the best candidate, was introduced in a quartz cuvette with an 808 nm continuous laser irradiation (Focus light, China) (0.5 W cm^−2^) for 15 minutes at room temperature (296 K). The temperature was recorded every 15 seconds with a digital thermometer using a thermocouple probe (Hanna Instruments, USA). Under continuous irradiation, the linear temperature increases as a function of time leading to the maxima of Δ*T* = 9.8 °C at 0.5 W cm^−2^ for BIOT-NFL-PEG-AuNPs.

MIA PACA-2 and F98 cells were seeded at a density of 200 000 cells per mL in 25 cm^2^ culture flasks and incubated at 37 °C and 5% CO_2_. Then, the cells were seeded in 96-well plates at 100 μL at 4000 cells per well and left for 24 and 48 hours. Then, 50 μL of the medium was removed and replaced by the colloidal solution and incubated for 24 hours. The medium was then removed, and the cells were washed three times with PBS (phosphate buffered saline) to remove excess non-internalized nanoparticles. Each well of the plate was irradiated by using an 808 nm laser source with a power of 0.5 W cm^−2^. The studies were conducted at two times: 5 and 10 minutes, to evaluate the impact of the “time” parameter on the results. After irradiation of the cells at 808 nm near infrared laser radiation, the medium was changed and left for 24 hours; then, a cell viability test was performed to verify that the nanoparticles had the same effect on the cells before and after the irradiation treatment.

### Dynamic light scattering (DLS) and zeta potential measurements

The size and zeta potential measurements were performed using a Zetasizer Nano ZS (Malvern Instruments, Malvern, UK) equipped with a He–Ne laser (633 nm, a fixed scattering angle of 173°) at room temperature as described previously.^23^

### Loading efficiency

The loading efficiency was calculated as follows under specific experimental conditions^20–22^ (eqn (1)):1
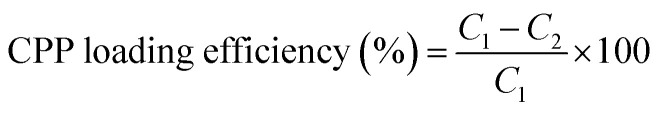
where *C*_1_ is the initial drug content, and *C*_2_ is the amount of the free peptide in the filtrate after separation of the nanoparticles.

### Stability of biotinylated-CPP-PEG-AuNPs (BIOT-NFL-PEG-AuNPs, BIOT-VIM-PEG-AuNPs, and BIOT-TAT-PEG-AuNPs)

The stability of all peptide conjugated nanoparticles was determined by recording the UV-visible spectra following their production and then checked 2 and 12 months later (Fig. S2, ESI†).

### Impact of nanoparticles on pancreatic cancer and glioblastoma cell lines (MIA PACA-2; F98)

Two cell lines obtained from the American Tissue Culture Collection (ATCC) were used in this study: MIA PACA-2 cells which are a human pancreatic cancer cell line and F98 cells which is a rat glioblastoma cell line. Both cell lines were cultured in Dulbecco's modified Eagle's medium (DMEM; Sigma-Aldrich) containing GlutaMax and supplemented with 10% of fetal bovine serum (Sigma-Aldrich), 1% of antibiotics (100× streptomycin/penicillin; BioWest, Nuaille, France) and 1% of non-essential amino acids (Sigma-Aldrich).

### Analysis of cellular internalization by transmission electron microscopy (TEM)

The cells (MIA PACA-2 or F98) were seeded in 12-well plates at 100 000 and 200 000 cells per well and were incubated for 24 hours at 37 °C and 5% CO_2_. Then, cells were treated with the particles. For BIOT-NFL-PEG-AuNPs, treatment was applied at 500 μM for 72 hours. For BIOT-TAT-PEG-AuNPs and BIOT-VIM-PEG-AuNPs, cells were treated at 250 μmol L^−1^ for 24 hours. For each experiment, PEG-AuNPs was used at the same concentration and at the same processing time. After incubation, cells were washed with 0.1 M phosphate buffer at pH 7.4 and fixed overnight at 4 °C with a solution of 2.5% glutaraldehyde in 0.1 M phosphate buffer. The next day, the fixator was removed, and cells were rinsed with 0.1 M phosphate buffer. Then, cells were rinsed with distilled water and post-fixed with a solution of 1% osmium tetroxide in water for 1 hour. After, cells were rinsed with water (3 times, 5 minutes) and incubated for 15 minutes in 50° ethanol, 15 minutes in 70° ethanol, 15 minutes in 95° ethanol and finally 3 times for 30 minutes in 100° ethanol. Then, cells were placed in a solution of 50% 100° ethanol and 50% Epon resin mixture (volume/volume) overnight. The next day, the Epon was removed and replaced by a pure Epon bath for 4 hours; then this bath was replaced by another pure Epon bath at 37 °C and 24 hours, at 45 °C and 72 hours and at 60 °C. When the resin has polymerized at 60 °C, ultra-fine sections of 60 nm thickness were made with a UC7 ultramicrotome (Leica, Wetzlar, Germany) and deposited on 150 mesh copper grids. The sections were contrasted with a solution of 3% uranyl acetate in 50° ethanol for 15 minutes and then rinsed with ultrapure water. The samples were observed using a 120 kV Jeol JEM-1400 electron microscope (Japan) with a SC1000 Orius model 832 (Gatan) 4k CCD camera.

## Conclusions

In our study, we designed and realized a new class of hybrid peptide-nanovector based on biotinylated cell penetrating peptides (BIOT-NFL, BIOT-VIM, and BIOT-TAT-peptides) through complexation with gold salt (Method IN). The success of this chemical methodology was confirmed by analytical techniques (Raman, UV-Vis, DLS, zeta potential, and TEM). We also investigated the chemical stability after several months and the biological interaction/penetration in pancreatic (PDAC, MIA PACA-2) and glioblastoma (GBM, F98) cancer cells with good and promising results in phototherapy applications. The design and assessment of these CPP hybrid gold nanoparticles as nanovectors can open new and promising perspectives in drug-delivery and cancer therapy.

## Author contributions

Celia Arib: methodology, formal analysis, investigation, validation, and writing-original draft preparation. Audrey Griveau: formal analysis, investigation, resources, and visualization. Joel Eyer: writing review and editing, visualization, supervision, and project administration. Jolanda Spadavecchia: conceptualization, methodology, writing review and editing, visualization, supervision, and project administration. All authors have read and agreed to the published version of the manuscript.

## Conflicts of interest

The authors declare no conflict of interest.

## Supplementary Material

NA-004-D2NA00096B-s001
