# Supplementary material for: Cell penetrating peptide (CPP) gold(iii) – complex – bioconjugates: from chemical design to interaction with cancer cells for nanomedicine applications
Source: Nanoscale Adv. 2022 May 12;4(14):3010–22. doi: 10.1039/d2na00096b (PMC9417459; doi:10.1039/d2na00096b)
Supplement: NA-004-D2NA00096B-s001 [file NA-004-D2NA00096B-s001.pdf]

Electronic Supplementary Material (ESI) for Nanoscale Advances.
This journal is © The Royal Society of Chemistry 2022

SUPPORTING INFORMATION

Cell Penetrating Peptides (CPP) gold (III) - complex - bioconjugates: from Chemical Design to interaction with cancer cells for Nanomedicine Applications

Celia Arib[1], Audrey Griveau[2], Joel Eyer[2*] and Jolanda Spadavecchia[1]

[1] CNRS, UMR 7244, CSPBAT, Laboratoire de Chimie, Structures et Propriétés de Biomatériaux et d'Agents Thérapeutiques Université Paris 13, Sorbonne Paris Cité, Bobigny, France .

[2] Laboratoire Micro et Nanomedecines Translationnelles, Inserm 1066, CNRS 6021, Institut de Recherche en Ingénierie de la Sante, Bâtiment IBS Institut de Biologie de la Sante, Université Angers, Centre Hospitalier Universitaire, Angers.

NFL-TBS.40-63

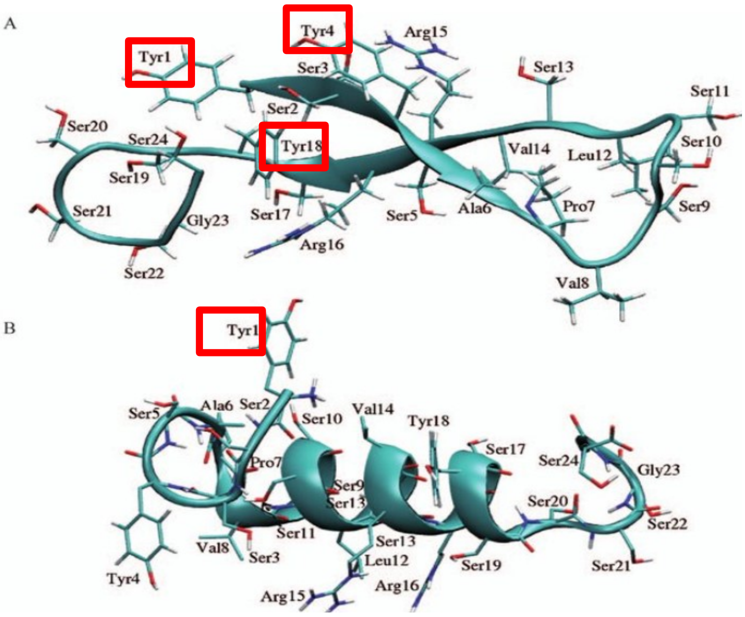

VIM

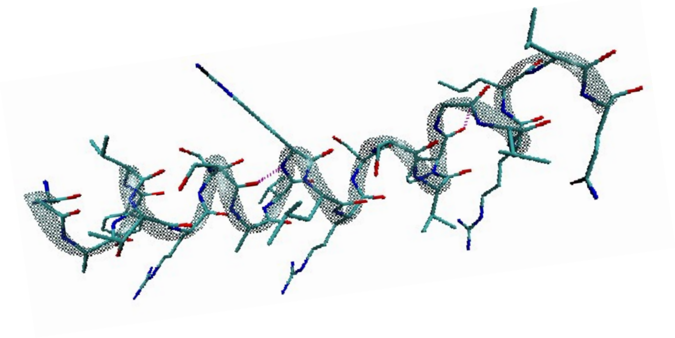

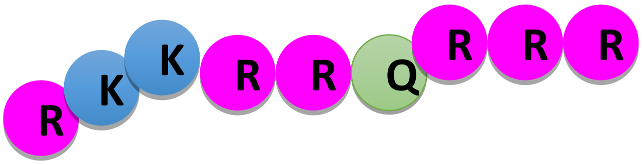

Arg Lys Lys Arg Arg Gln Arg Arg Arg

TAT

Figure S1: Schematic structures of NFL-TBS.40-63, VIM and TAT-peptides as previously described in literature[29,30,36].

.

A)

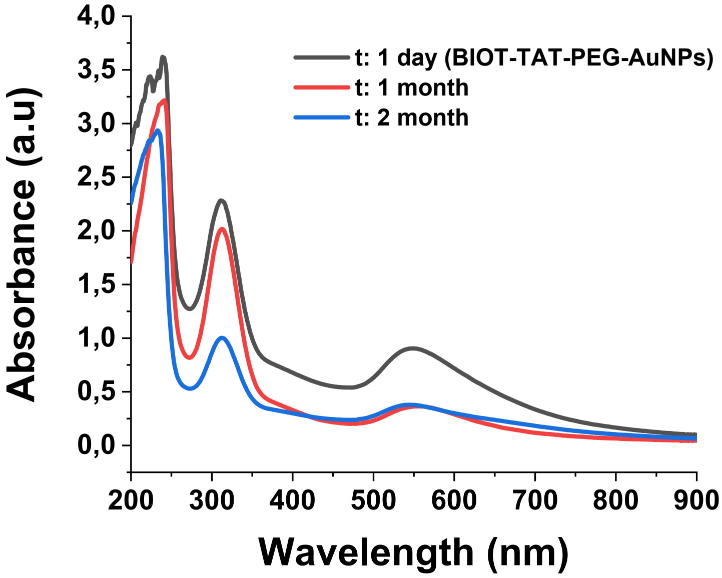

B)

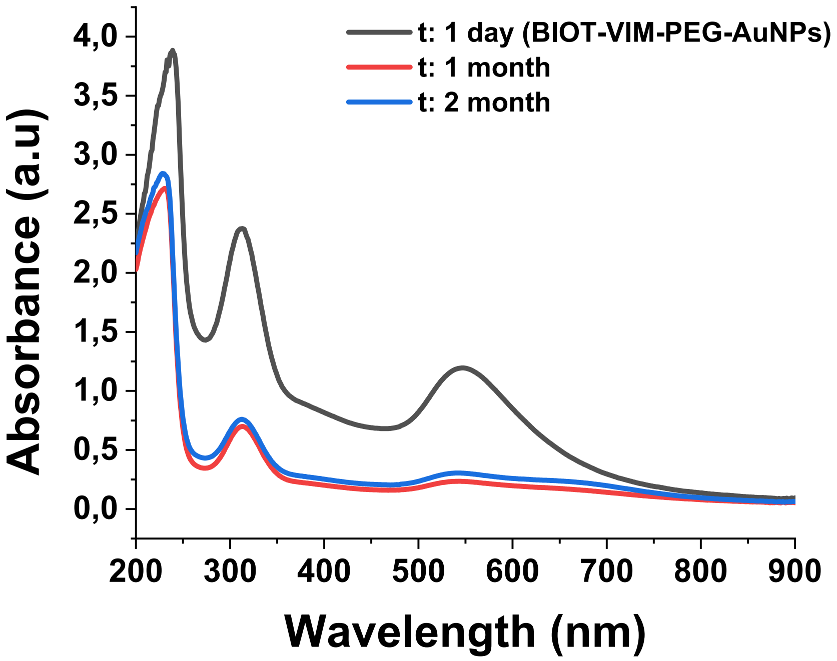

Figure S2: UV-Vis spectra of A) BIOT-TAT-PEG-AuNPs and B) BIOT-VIM-PEG-AuNPs under experimental conditions a t: 1 day (grey line) and after 2 months (blue line).

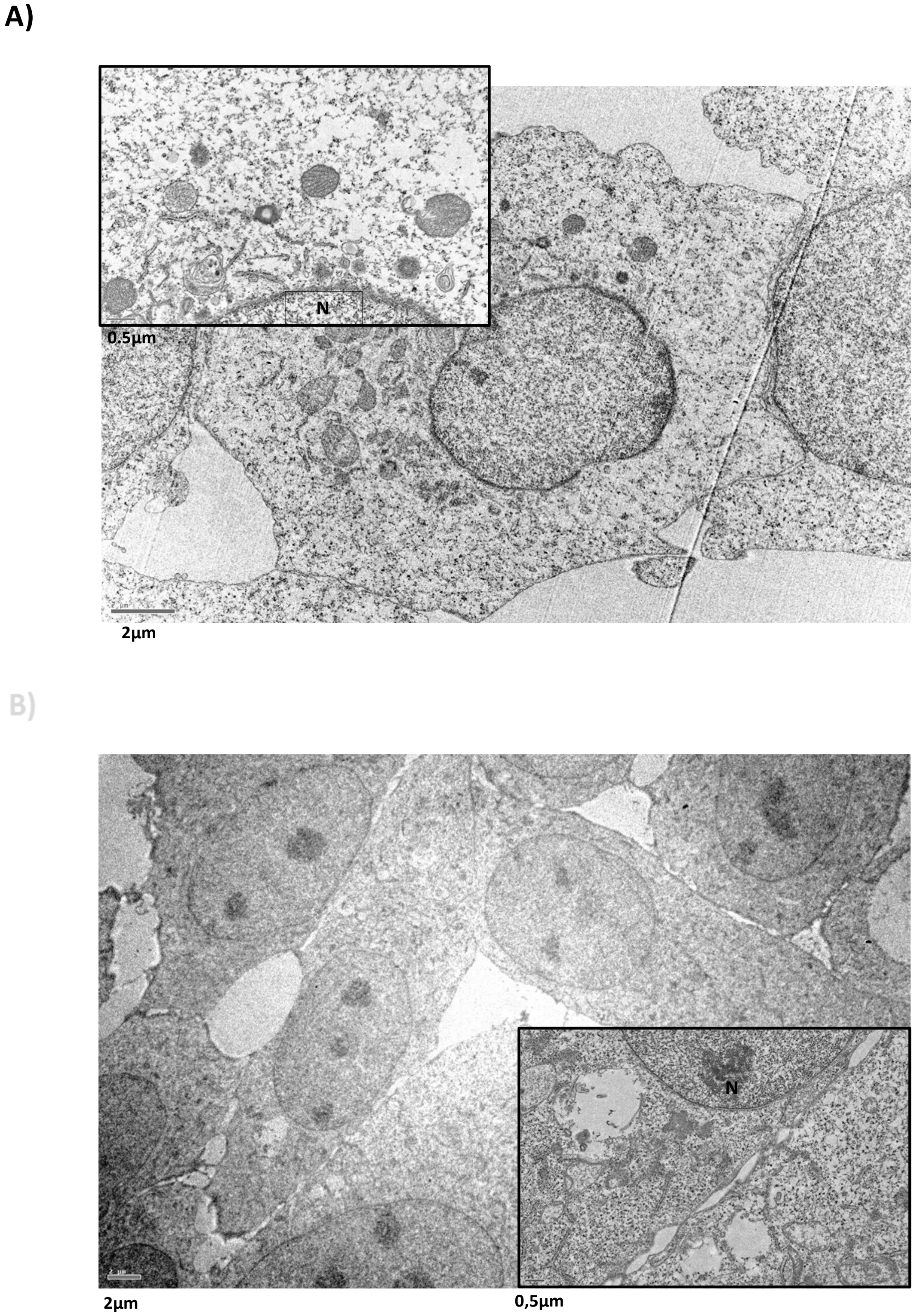

Figure S3: Transmission electron microscope (TEM) images of **A)** Mia PACA-2 and **B)** F98 cells as negative control (NT). N for nucleus.

A)

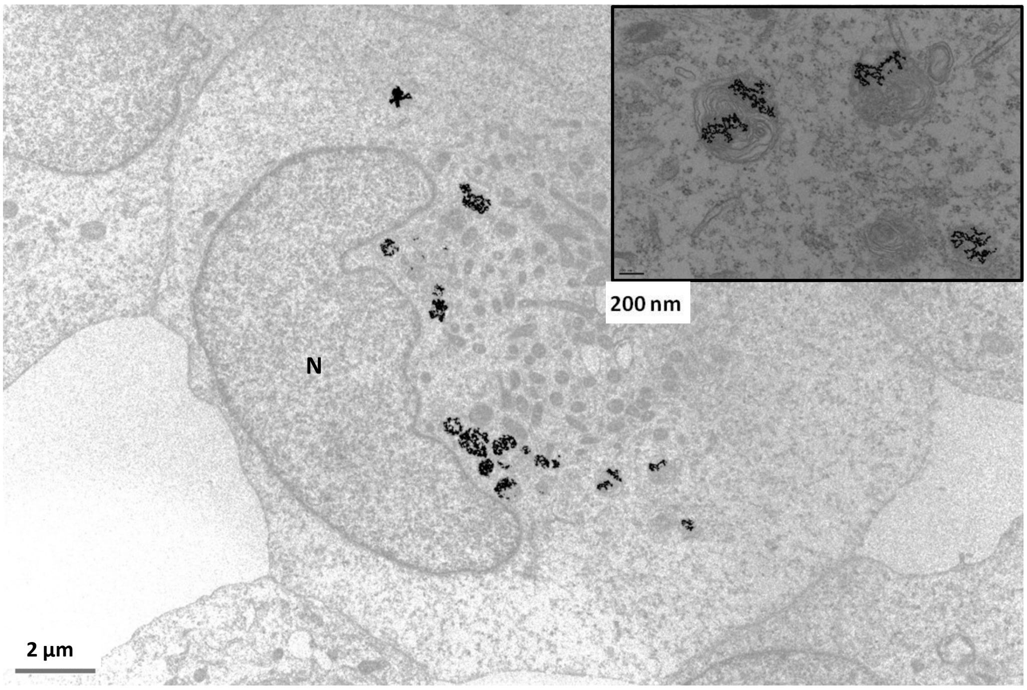

B)

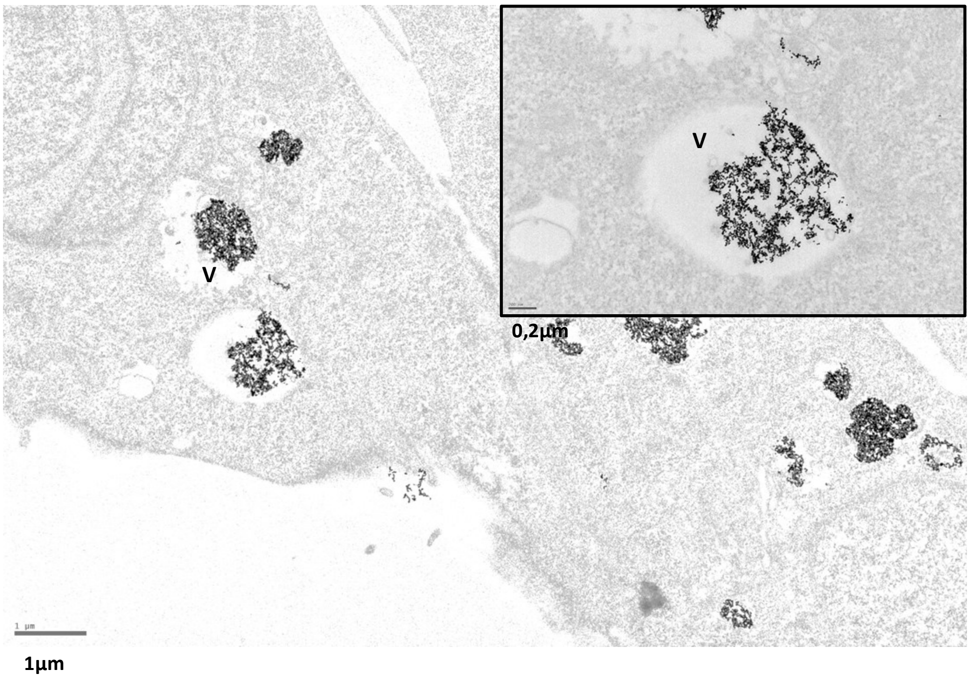

Figure S4: TEM images of **A)** Mia PACA-2 and **B)** F98 cells treated with PEG-AuNPs at 500 µmol/L for 72 hours. N for nucleus and V for vacuoles.
